# Changing patterns of meat consumption and greenhouse gas emissions in Australia: Will kangaroo meat make a difference?

**DOI:** 10.1371/journal.pone.0170130

**Published:** 2017-02-14

**Authors:** Shyama Ratnasiri, Jayatilleke Bandara

**Affiliations:** Department of Accounting Finance and Economics, Griffith University, Brisbane, Queensland, Australia; Agricultural University of Athens, GREECE

## Abstract

The Australian per capita consumption of ruminant meat such as beef and lamb has declined over the last two decades. Over the same period, however, per capita consumption of non-ruminant meat such as chicken and pork has continued to increase. Furthermore, it is now observed that the human consumption of kangaroo meat is on the rise. This study investigates the implications of these changes in meat consumption patterns on Green House Gases (GHGs) emission mitigation in Australia using a Vector Auto Regression (VAR) forecasting approach. Our results suggest that the increase will continue in non-ruminant meat consumption and this will not only offset the decline in ruminant meat consumption, but will also raise the overall per capita meat consumption by approximately 1% annually. The per capita GHGs emissions will likely decrease by approximately 2.3% per annum, due to the inclusion of non-ruminant meat in Australian diets. The GHGs emissions can further be reduced if the average Australian consumer partially replaces ruminant meat with kangaroo meat.

## Introduction

Even though reducing Greenhouse gases (GHGs) has been discussed around the globe for more than four decades with international bodies establishing several mitigation protocols as an outcome, the GHGs emitted by humans are still increasing every year. What this suggests is that mitigation efforts so far have not been able to deliver the desired outcome of reducing GHGs. This, in turn poses the question of whether we should be looking to explore avenues that have not been explored before to mitigate GHGs emissions. What has been explored so far is central to supply-side or production systems, with components such as technological or biological improvements, and supply management strategies that includes emissions targets and taxes. Until recent years very little was explored about demand-side components such as changes in consumer behaviour, socio-cultural and economic values, risk taking attitudes and the ways in which they can affect the GHGs emissions mitigation. This study attempts to contribute to this strand of literature by exploring changes in consumer behaviour, essentially dietary changes over time and their potential implications for GHGs emission reduction.

The notion that dietary changes might play a role in GHGs emission mitigation is relatively new in related literature. In 1997 Goodland revealed that diet is an important consideration in fostering environmental sustainability [1]. Since then, a number of studies have recognised that dietary changes hold a large mitigation potential due to considerable variation in the GHGs intensity per unit of food in different food groups [2–6]. Animal based food generally having greater emissions than vegetable products owing to GHGs (methane) produced in enteric fermentation of ruminants and inefficiencies in growing cereals crops to feed livestock [7–10]. For this reason, it is well accepted that dietary changes towards less meat oriented diets could significantly reduce food related GHGs emissions [4, 11–19]. To facilitate this shift to less meat, two credible paths can be suggested. The first is substitution of meat by plant oriented diets [20, 21]. The second is to substitute high GHG intensive food items (e.g beef, lamb) with low GHG intensive products (pork, chicken, ocean fish) which still accounts for nutritional requirements and dietary preferences and forgoes the need to totally shift away from meat [1, 12, 22].

Although substantial reductions could be achieved from both these options, one important issue to consider is who should moderate meat consumption. Over the past two decades, population growth coupled with an increase in meat consumption in per capita terms has resulted in an increase in overall global meat consumption. It is expected that this tendency will continue even stronger in developing countries due to their rapid economic growth [23–26]. In developed nations such as Australia UK, USA and EU region however, there has been a tendency to reduce red meat (beef and lamb) consumption. This is despite having very high per capita consumption levels already[27, 28]. Owing to these trends, some researchers suggest targeting consumers with very high per capita consumption in the developed world would be more effective, while others propose to include developing countries as well due to their huge population [15, 29].

Drawing from the mitigation potential of dietary changes, various demand management policies have also been advocated in the recent literature. One suggestion is to advocate demand led emission mitigation policy instruments such as GHG weighted consumption taxes on animal food products, akin to the idea presented in [1]. In similar studies, Wirsenius et al[30] for European Union, Edjabaou and Smed [31] for Denmark and Briggs et al [32] for the UK conclude that this type of consumption tax would significantly reduce food related GHG emissions. Other researchers have explored ways of assisting consumers in making environmental friendly dietary choices [33, 34] and constructing pathways towards meat substitution [35].

## Motivation

**Fig 1:**
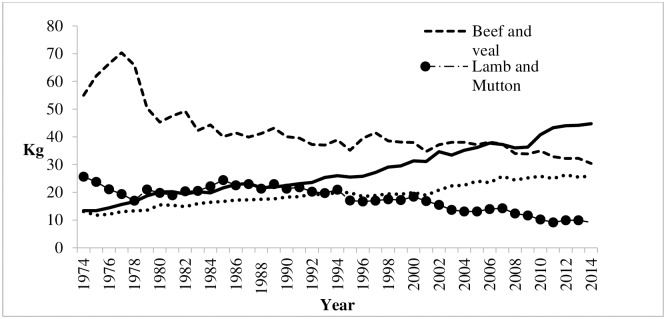
Per capita meat consumption in Australia 1974–2014. (Source ABARE, 2016).

We explore the option of dietary changes in emissions mitigation in the context of Australian meat consumers in this study. The motivation is due to a recent observation that there has been significant change taking place in meat consumption patterns among Australian consumers. It has been observed that the Australian consumption of ruminant meat, such as beef and lamb, has declined over the last two decades. For example, as depicted in Fig 1, per capita beef and lamb consumption fell by approximately 45% and 64% respectively between 1974 to 2014 [36]. Meantime, per capita consumption of non-ruminant meat such as chicken and pork continued to increase over the same period [36]. In particular, per capita chicken consumption has tripled while per capita pork consumption doubled during the same period [36]. Furthermore, it has been observed that human consumption of kangaroo meat has increased tremendously, with many supermarkets now having a designated section for kangaroo meat products. Although in the past kangaroo meat has been used as a pet food, the demand for human consumption has increased following the recognition that kangaroo meat is as a lean red meat substitute. Taken together, it is apparent that the consumption patterns of different meat products have changed significantly in recent years in Australia.

Besides the changing consumer behaviour, the other significant component of the question is the potential these changes have to influence GHGs emissions mitigation in Australia. To this end, it is important to recognise that current GHGs emissions in the Australian livestock sector, which provide almost the entire meat requirement of the population, are significantly high. Livestock emissions are one of the major sources of GHGs emissions in Australia, accounting for approximately 12 per cent of Australia’s total GHGs emissions or 70 per cent of agriculture emissions—more than triple the global average [37, 38]. These livestock emissions come mainly from the enteric fermentation that takes place in the digestive system of ruminants such as cattle, buffalo, sheep, and goats. Research that investigates possible alternatives with the potential to reduce GHGs from the ruminant livestock sector is therefore of paramount importance.

In terms of research that has been undertaken to explore alternative solutions to mitigate livestock emissions most of them focus on the biological aspects of this question. For example these include the selective breeding of livestock that emit lower levels of methane, or exploring alternative feed options that reduce methane production in enteric fermentation. However, changes in consumption patterns could also be a viable alternative to mitigate GHGs in livestock sector even though this is not well accepted by some researchers. The Australian meat consumption data in past three decades suggests that consumers have reduced their *ruminant meat* consumption, and increased *non-ruminant* livestock consumption.

It is acknowledged that the impacts of such an alternative on the livestock sector, as well as on the overall economy, can be critical. However, a partial replacement of ruminant meat consumption by other species which produce none or relatively less methane in their enteric fermentation processes is still an arguably robust strategy for abating GHGs. In identifying which non ruminant meat products can be substituted, meats such as chicken and pork have already been in the diet for Australian consumers. Even though kangaroo meat is included in the diet recently, kangaroo researchers advocate this meat has the highest potential to influence GHG mitigation as kangaroos do not produce GHGs in their digestion process.

In light of these suggestions, there has been a debate in recent years over the issue of kangaroo harvesting on moral and scientific grounds [39–41]. While the proponents of kangaroo harvesting argue that it minimises negative effects of kangaroos on farm outputs, others oppose it on the moral grounds of the kangaroo being Australia’s national icon [42]. Although there has been a long history of advocating for kangaroo harvesting to solve Australian rural farming problems, in particular, land degradation due to overgrazing in rangelands and by looking at kangaroos as pests (see for example, [39]), there has not been much work examining the link between kangaroo meat substitution for other meat and the reduction of GHGs emission. The exception to this is the work of Wilson and Edwards [43]. Using a simple spreadsheet model, Wilson and Edwards [43] demonstrated that the GHGs savings Australia could make would be 3% of its annual emissions if livestock were reduced only on the rangelands, and kangaroo numbers were increased to produce the same amount of meat. Studies supporting the harvesting of kangaroos for reasons related to both the traditional arguments as well as the basis of reduction in GHGs, have also been challenged by opponents of kangaroo harvesting in recent years (example, [40]). There is clearly a need for further systematic studies to act as inputs in this debate.

Against the above background, the main objective of this study is to investigate the changing pattern in consumption of different types of meat in Australia and evaluate the potential this has to mitigate future GHGs emissions. By considering these questions, the study intends to make a number of innovative contributions. Firstly, the changing pattern of meat consumption over time due to long run variations in economic factors will be examined to explore the future trends in GHGs emissions in Australia. This type of study has not been undertaken relevant to Australia despite the existence of a large body of literature on demand elasticity estimates for Australian meat products (see [44] for a survey). Secondly, the study will estimate the level at which non-ruminant meat can reduce future GHGs emissions. A simple econometric technique will be employed to examine the changes in long-term meat consumption patterns and to forecast expected changes in GHGs emissions. Thirdly, the study will estimate the reduction in GHGs if ruminant meat is partially replaced by non-ruminant meat such as kangaroo meat. We expect this study to provide further evidence to support the idea that increasing the consumption of non-ruminant meat (e.g. kangaroo) in the Australian diet can make a difference in terms of reducing GHGs. To this end, the study provides important policy insights in relation to two major economic issues in Australia. The first is related to Australia’s commitment to reduce GHGs according to the Kyoto protocol targets. The second is related to Australia’s widespread rangelands management. For an effective use of rangelands, policies need to be reformulated to consider kangaroos as an economic resource that produce lean meat with low cholesterol for an emerging market, as opposed to pests in graze lands.

## Theoretical framework

As noted previously, the key objectives of this empirical study is to explore the future patterns of meat consumption in Australia based on past experience and subsequent changes in GHGs emissions. For this purpose a forecasting exercise will be carried out using a simple Vector Auto Regression (VAR) model that entails estimation of a system of equation in which both prices and quantities are determined endogenously. The choice of the VAR considers forecast performance out of the sample. This is despite there being a large volume of literature in relation to consumer demand estimation including the Linear Expenditure System [45], the Rotterdam model [46, 47] the Almost Ideal Demand System (AIDS) [48] and their variants such as the models in [49–53]. Wang and Bessler [54] present a comprehensive discussion on forecasting approaches for meat demand.

We start with the following basic specification for consumption (*C*) in each type of meat, which is a function of own price (*P*_*i*_), prices of other meats (*P*_*j*_) and Income (*I*).

$$C_{ti}=f(P_{it},P_{jt},I_{t})$$ (1)

Here subscript *i* denotes the type of meat we are interested in estimating the demand relationship for, *j* = 1…*n*, *j* ≠ *i* where *n* is the types of meat consumed and *t* is the time period.

The estimations involving aggregate demand and supply often lead to a simultaneity problem. This is particularly evident in the analysis of meat consumption. The simultaneity problem can however be circumvented by a multivariate VAR model if all variables are having unit roots. We employ a multivariate VAR to explore the relationship between these variables. We use a nine dimensional form of the VAR model which is expressed in the following equation:

$$X_{t}=\phi_{1}X_{t-1}+\ldots \ldots .+\phi_{k}X_{t-k}+\varepsilon_{t}\;t=1,\ldots \ldots ,T.$$ (2)

Here, *X*_*t*_ is the non-stationary vector where, *X*_*t*_ = [∑*C*_*it*_, ∑*P*_*it*_, ∑*I*_*t*_] as denoted above, *ϕ*_1_…..*ϕ*_*k*_ are coefficient matrixes and *ε*_*t*_ is the vector of error terms. The number of lags is denoted by the notation *k*. We consider the maximum lag length of three depending on the sample size. Our estimation used lag length of one. The method used to choose the appropriate lag length involves Akaike information Criterion and (AIC) and Schwartz’s Criterion (SC) [55, 56].

## Data and estimation

In order to estimate the above model, data on consumption (*C*_*it*_) of different meat types, prices (*P*_*it*_ and *P*_*jt*_) of different meat products, and per capita income (*I*_*t*_) of the consumers were collected for the period 1968–2011. The data on per capita consumption (*C*_*it*_) of beef, lamb, pork and chicken and their prices (*P*_*it*_) were obtained from Agricultural commodity statistics (2012) published by ABARE. Per capita income (*I*_*t*_) data was also obtained from time series spread sheets published by Australian Bureau of Statistics (ABS). In our analysis, for a meat type *‘i’* in consideration, we use the prices of remaining meat types (i.e when *i≠j*.) as cross prices (*P*_*jt*_) in the models estimated.

Estimation of VAR requires the variables to be tested for stationarity of the data. We employed the two tests of; Augmented Dickey-Fuller test [57] and Phillips-Perron test [58]. An appropriate multivariate VAR model was estimated based on stationarity properties of data. Based on VAR estimates, future meat consumption is then forecasted. This model would be perfect for our purpose if kangaroo meat consumption was also endogenously determined. The non-availability of data imposes a methodological limitation in the empirical estimation however we assumed a more general, simple linear trend to forecast future kangaroo meat consumption using data from Rural Industries Research and Development Corporation (RIRDC) of Australian Government [59].

## Results and discussion

The properties of data and empirical results obtained from the model are presented and evaluated in this section. In Table 1 we present the results of unit root tests used to test stationarity of data. In this table, all *C* variables are related to per capita consumption of different types of meat, all *P* variables are related to different prices and *I* is per capita income. It is evident from the results shown in Table 1, that all variables are non-stationary at their level forms, however, they are stationary (i.e. *I(1)*) after the first difference. Therefore the above the VAR model was estimated in the first difference forms of the variables.

**Table 1:** Results of unit root test.

Variables	Augmented Dickey-Fuller test statistic				Phillips-Perron test statistic			
	Level		First Difference		Level		First Difference	
	Intercept	Intercept and Trend	Intercept	Intercept and Trend	Intercept	Intercept and Trend	Intercept	Intercept and Trend
*C(pork)*	-0.4321	-2.7780	-6.8222***	-6.7376***	-0.6116	-2.8864	-6.8260***	-6.7418***
*C(chicken)*	-0.6181	-3.0774	-7.6498***	-7.5344***	-0.6810	-3.1287	-8.8726***	-8.7211***
*C(beef)*	-1.3816	-2.0741	-4.4102***	-3.0195	-1.7214	-2.2781	-4.4107***	-4.4182***
*C(lamb)*	-2.4347	-2.5005	-5.8807***	-5.9084***	-2.5060	-2.4585	-5.8819***	-5.9077***
*P(pork)*	1.5811	-1.3058	-5.2236***	-5.4535***	1.4977	-1.4090	-5.2271***	-5.4014***
*P(chicken)*	-0.7950	-1.4724	-5.1997***	-5.2078***	-0.8244	-1.6805	-5.1997***	-5.2104***
*P(beef)*	0.1804	-2.8745	-4.0726***	-4.0952**	0.7798	-2.1664	-3.9629***	-3.9200**
*P(lamb)*	0.9800	-1.4246	-4.0039***	-4.2987***	1.9584	-1.1184	-3.9868***	-4.3092***
*I*	2.0293	-0.3461	-4.4517***	-5.0388***	2.0262	-0.4016	-4.4236***	-5.3823***

*** denotes rejection of null hypothesis at 1%,

**denotes rejection of null hypothesis at 5%,

* denotes rejection of null hypothesis at 10%.

**Fig 2:**
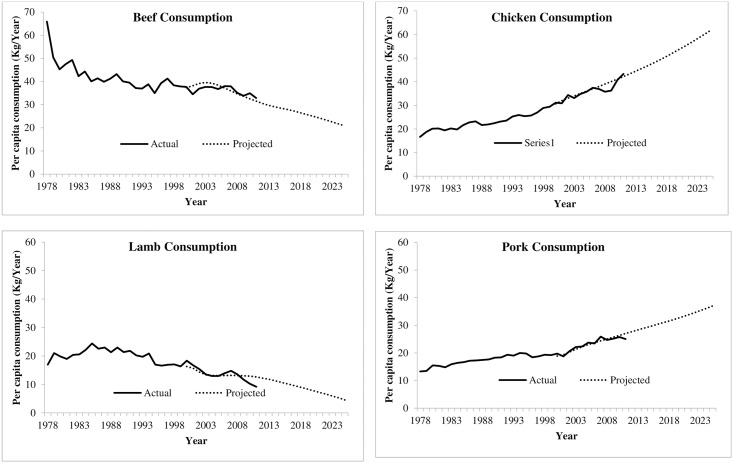
Actual and predicted per capita meat consumption quantities of beef, lamb, pork and chicken.

Fig 2 illustrates the estimated future meat consumption in Australia for beef, chicken, lamb and pork using the modelling approach. It is evident from the Fig 2 that the beef and lamb consumption in Australia is predicted to decline over time while pork and chicken consumption is predicted to increase. Table 2 also summarizes forecasts for per capita consumption of meat for only two future years. For example, the number in Table 2 column 2 shows that forecasted Australian per capita beef consumption in 2020 is 24.96 kgs and this will be decrease to 20.83 kgs in 2025. Considering these outcomes in Fig 2 and table together, it is predicted that the consumption of ruminant meat that emits significant amount of GHGs will continue to decrease in Australia while non-ruminant meat consumption will increase.

**Table 2:** Forecasts for per capita meat consumption (kg).

Year	Beef	Chicken	Lamb	Pork
2020	24.96	53.65	7.81	33.00
2025	20.83	61.55	4.57	37.03

The corresponding forecasted quantities of GHGs emissions are presented in Table 3 below. The conversion factors used in the estimates are obtained from Fiala[60] and Edwards-Jones et al.[61]. We use the carbon dioxide equivalent quantities (kg) emitted from one kilogram of production of beef, chicken, pork and lamb which are 14.8, 1.1, 3.8 and 10.1 respectively in our calculations.

**Table 3:** Forecasts for per capita GHGs emissions (kg of CO2 equivalent).

Year	Beef	Chicken	Lamb	Pork
2020	369.47	59.01	78.92	125.39
2025	308.34	67.70	46.18	140.70

As apparent from Table 3, the GHGs emissions from ruminant meat consumption are projected to decrease in Australia, whilst the GHGs emissions from non-ruminant meat consumption are projected to increase. This increase in GHGs emissions is expected to offset the decline due to ruminant meats, thus, producing a net decreasing effect.

It is interesting to explore the overall impact of these changes on meat consumption patterns over future years, and how this translates into overall GHGs emissions. The results forecasting this total meat consumption (i.e the sum of four different meat types under consideration) and the sum of associated GHGs emissions in Australia, are presented in Table 4. In this calculation, 2010 is considered as the base year. As is evident from Table 4, the future total meat consumption is projected to increase in a gradual manner. More precisely, according to the forecasts, per capita meat consumption in Australia is expected to increase by approximately 1% per annum over the period from 2010 to 2025. In contrast, GHGs emissions associated with meat consumption are expected to decline considerably during the same period. According to these forecasts the GHG emissions are expected to decline by nearly 2.3% per annum. These empirical findings suggest that even though the total per capita meat consumption is expected to increase in Australia during the next decade, the *composition* of meat consumption is expected to change significantly. That is, the patterns observed over time suggest that consumers have been substituting more ruminant meat (beef and lamb) with non-ruminant meat such as pork and chicken. If this trend continues, the future overall GHGs emissions from meat consumption will tend to decline. This is an interesting finding to emerge considering that the GHGs emission reduction is one of the national priorities in Australia.

**Table 4:** Forecasts for total meat consumption and associated GHGs emissions.

Year	Total meat consumption (kg per capita)	Total GHGs emissions (kg of CO2 equivalent)
2020	119.42 (11.7%)	791.45(-25.7%)
2025	123.98 (15.9%)	767.39 (-33.9%)

Figures within parenthesis show the percentage change in the predicted figure from the variable’s corresponding 2010 actual observation.

Given that future GHGs from meat consumption has a declining trend, it is also important to explore the other possibilities advocated by scientists in line with the Australian trend observed in the motivation section of this paper. That is, the possibility of partial replacement of red meat by another red meat that entails small or no GHGs in its production process. To that end, the remainder of this section will focus on analyzing a case that involves the partial replacement of red meat consumption by recently popularized kangaroo meat in Australia. Table 5 presents the projected per capita human consumption of kangaroo meat in column two and the resulting GHGs emissions from such consumption in column three. To convert kangaroo meat quantities into GHG emissions in this study, a reasonable estimate of the GHG emissions from kangaroo is required. Such a figure is yet to be confirmed in the literature although it has been well accepted that kangaroos emit much less GHGs, particularly methane (CH_4_), than that of ruminants [62–64]. In our calculations we use 1.30 CO_2_ equivalents for one kilogram production of kangaroo meat which is an average of the estimates reported in the literature (see [43, 62–64]. This figure is also conceivably realistic, assuming the average weight of a kangaroo is 25 kilograms (kg) and lifespan is 10 years despite some claims that farming kangaroos can account more for GHGs than harvesting from range lands (See also [39, 65, 66].

**Table 5:** Forecasts for per capita human consumption of kangaroo meat and expected reduction in GHGs emissions.

Year	Kangaroo meat consumption (kg)	GHGs emissions from kangaroo meat (kg of CO2 equivalent per capita)
2020	0.77	1.01
2025	0.99	1.29

In this analysis we were also interested in exploring the changes in future GHG emissions if Australians replace their current meat consumption with kangaroo meat. Drawing on the estimated increases in kangaroo meat consumption, however, it can be seen that kangaroo meat can only partially, rather than totally, replace Australia’s other meat consumption. In the context of future policy implications, we can now consider a policy scenario in which Australian consumers replace their “red” meat (beef and lamb) consumption with kangaroo meat. This is a more realistic assumption given that kangaroo meat is predominantly accepted as an alternative type of a red meat [67]. Particularly in food and meat science literature, kangaroo meat is identified as a red meat substitute (see survey by Barai et al[68].

When analyzing this policy scenario, the percentage of each type of red meat replaced by kangaroo meat is calculated based on forecasted kangaroo meat consumption as follows. If forecasted per capita kangaroo meat consumption in future ‘*t*^*th*^’ year is $C_{kt}^{*}$ and the same figure exists for one of the red meats, for example beef is $C_{bt}^{*}$, we assume that $C_{kt}^{*}/C_{bt}^{*}$ proportion of beef is replaced by kangaroo meat. Using this, the percentage of the red meat replacement varies over the years. We therefore explore two scenarios separately as follows;

*Scenario I*: The estimated increase in kangaroo meat consumption partially replaces only beef
*Scenario II*: The estimated increase in kangaroo meat consumption partially replaces only lamb

**Fig 3:**
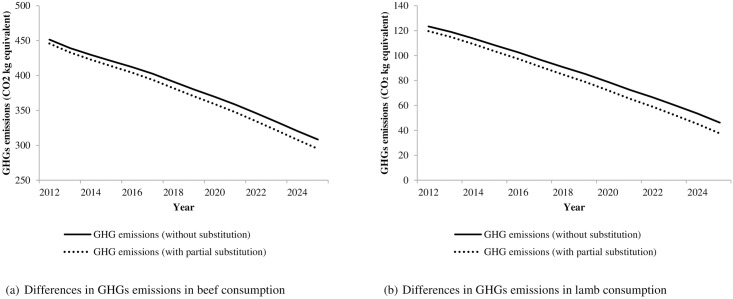
Projected reduction in GHS emissions due to substitution of red meat by kangaroo meat.

Table 6 presents the forecasted reduction in GHGs emissions under the two scenarios discussed above. Under Scenario I, the partial replacement of beef by kangaroo meat will *further* reduce per capita GHGs by 10.38 kg of CO2 equivalent per capita in 2020. When comparing this with the previous level of estimated GHGs emissions (without substitution) (Table 3) this is a further reduction of approximately 2.8%. A similar kind of explanation will apply to Scenario II. In the case of Scenario II, kangaroo meat consumption in lieu of consumption of lamb will *further* reduce per capita GHGs emissions and this reduction will be approximately 8.6% in 2020. Fig 3 provides a supplementary illustration of the reduction in GHGs emissions over future years for two scenarios.

**Table 6:** Reduction in GHGs emissions due to partial replacement by kangaroo meat.

year	Reduction in GHGs emissions (kg of CO2 equivalent per capita)	
	Scenario I	Scenario II
2020	10.38 (2.8%)	6.77 (8.6%)
2025	13.32 (4.3%)	8.68 (18.8%)

Figures within parenthesis are reduction in GHGs emissions expressed as a percentage of the variable’s corresponding non substitution case.

It is important to note that in this analysis we do not consider socio-economic and other variables that may affect kangaroo meat consumption. We conjecture that the effect of such variables will enhance kangaroo meat consumption estimates for the future. Increased kangaroo meat consumption may even reinforce the substitution effect resulting in further decreases in emissions. We therefore believe that our estimates are quite conservative. Still, our study predicts that the future per capita GHGs emissions resulting from meat consumption has a declining trend.

## Discussion and concluding remarks

This study has attempted to examine the link between changing patterns in meat consumption and GHGs emissions in Australia and the possibility of reducing GHGs further by encouraging substitution of kangaroo meat for beef and lamb. We observed that Australian consumers have been substituting a significant amount of ruminant meat with non-ruminant meat in the recent past. Our results suggest that, if such a trend persists, per capita ruminant meat consumption (that is beef and lamb consumption) will gradually decline over time. This will, in turn, translate into a significant reduction in per capita GHG emissions resulting from meat consumption.

The results also suggest that there will be an increase in non-ruminant meat (chicken and pork) consumption. This increase will not only offset the decline in ruminant meat consumption but will also raise the overall per capita meat consumption in Australia.

With regards to patterns in overall per capita meat consumption, the results suggest that it may increase by approximately 1% annually between the period 2010 and 2025. In addition to this, the per capita GHGs emissions will likely decrease over the same period with the possibility of an approximate 33% drop in per capita GHGs emissions due to the aforementioned changes in meat consumption patterns. This is equivalent to an average annual decrease of approximately 2.3%.

Finally, our study confirms that GHGs emission can further be reduced by the partial replacement of ruminant meat with kangaroo meat in a typical Australian diet. Thus, in addition to the decline in GHGs emissions due to the above changes in consumption patterns in recent years, our findings suggest that a further reduction of approximately 4.3% can be achieved by 2025 if beef is partially replaced with kangaroo meat. This figure is 18.8% for the same period, if an average Australian consumer partially replaces lamb with kangaroo meat.

In conclusion, our study suggests that Australian meat consumers are currently contributing to GHG emission reduction by changing meat consumption patterns. If such changes continue, the per capita GHGs emissions due to meat consumption will fall. The empirical results of this study have two important policy implications. The first falls in line with the Kyoto protocol targets of reducing GHGs emission reduction. Our results suggest that the increasing trend in non-ruminant meat consumption has a huge potential to reduce GHG’s and therefore, policies that enhance such economic behaviours are desirable in macroeconomic environments. The second policy implication is related to the problems associated with rangeland managements and effective utilization of rangelands. A policy measure can be formulated around the idea that kangaroo acts as an economic resource for an emerging market of lean meat with low cholesterol rather than a pest in rangelands that affect graziers’ productivity. Essentially, more research is needed on how this could be achieved without having negative implications on the rest of the livestock production and overall economy.

Finally, besides the major GHGs mitigation interventions currently in place, some modest actions / behavioural changes of individuals such as changing consumption of meat could bring considerable gains in abating GHG emissions around the globe. However, if this type of demand based solution is to become a reality an increased attention to associated socio-political and economic research is warranted.
